# Angiopoietin-1 is associated with cerebral vasospasm and delayed cerebral ischemia in subarachnoid hemorrhage

**DOI:** 10.1186/1471-2377-11-59

**Published:** 2011-05-26

**Authors:** Marlene Fischer, Gregor Broessner, Anelia Dietmann, Ronny Beer, Raimund Helbok, Bettina Pfausler, Andreas Chemelli, Erich Schmutzhard, Peter Lackner

**Affiliations:** 1 Department of Neurology, Innsbruck Medical University, Innsbruck, Austria; 2 Department of Radiology, Innsbruck Medical University, Innsbruck, Austria

**Keywords:** Subarachnoid hemorrhage, cerebral vasospasm, angiopoietin, delayed cerebral ischemia

## Abstract

**Background:**

Angiopoietin-1 (Ang-1) and -2 (Ang-2) are keyplayers in the regulation of endothelial homeostasis and vascular proliferation. Angiopoietins may play an important role in the pathophysiology of cerebral vasospasm (CVS). Ang-1 and Ang-2 have not been investigated in this regard so far.

**Methods:**

20 patients with subarachnoid hemorrhage (SAH) and 20 healthy controls (HC) were included in this prospective study. Blood samples were collected from days 1 to 7 and every other day thereafter. Ang-1 and Ang-2 were measured in serum samples using commercially available enzyme-linked immunosorbent assay. Transcranial Doppler sonography was performed to monitor the occurrence of cerebral vasospasm.

**Results:**

SAH patients showed a significant drop of Ang-1 levels on day 2 and 3 post SAH compared to baseline and HC. Patients, who developed Doppler sonographic CVS, showed significantly lower levels of Ang-1 with a sustained decrease in contrast to patients without Doppler sonographic CVS, whose Ang-1 levels recovered in the later course of the disease. In patients developing cerebral ischemia attributable to vasospasm significantly lower Ang-1 levels have already been observed on the day of admission. Differences of Ang-2 between SAH patients and HC or patients with and without Doppler sonographic CVS were not statistically significant.

**Conclusions:**

Ang-1, but not Ang-2, is significantly altered in patients suffering from SAH and especially in those experiencing CVS and cerebral ischemia. The loss of vascular integrity, regulated by Ang-1, might be in part responsible for the development of cerebral vasospasm and subsequent cerebral ischemia.

## Background

Subarachnoid hemorrhage (SAH) accounts for 2-5% of all new strokes and is still associated with high morbidity and mortality [1,2]. In about 85% of all patients, non-traumatic SAH is caused by the rupture of an intracranial aneurysm [3]. Cerebral vasospasm (CVS) is one of the most important complications of SAH and may be associated with delayed cerebral ischemia (DCI) frequently resulting in poor functional outcome and death [4-6]. Various mechanisms are discussed to be involved in the pathophysiology of CVS. Apart from smooth muscle contraction and an increase of spasmogens such as oxyhemoglobin or bilirubin oxidation products an imbalance of endothelium-derived vasoconstrictor and vasodilator substances is thought to play a crucial role in CVS pathogenesis [7,8].

Angiopoietin-1 (Ang-1) and -2 (Ang-2) are two antagonistic ligands on the endothelial Tie-2 receptor regulating vascular homeostasis and endothelial stability [9,10]. Ang-1 is constitutively expressed by perivascular cells such as smooth muscle cells, fibroblasts, pericytes, platelets or neutrophils [9]. Constitutive Ang-1/Tie-2 signaling is important for endothelial cell survival and the maintenance of vascular integrity [11]. Ang-1 mediates anti-inflammatory and anti-adhesive properties on the vascular endothelium and promotes interendothelial cell-cell stability directly antagonizing hyperpermeability mediated by vascular endothelial growth factor [12-14]. Ang-2 is almost exclusively expressed by endothelial cells and released upon endothelial activation [15]. Ang-2 has proapoptotic and proinflammatory effects on endothelial cells, promotes the expression of adhesion molecules facilitating leukocyte migration and induces vascular leakage [16,17].

High serum levels of Ang-2 together with a decrease of the protective factor Ang-1 are associated with poor outcome and death in acute lung injury, severe sepsis, cerebral malaria and various other diseases [18-24]. In a recent publication by our group, we showed that endothelial microparticles are elevated in patients with CVS and DCI indicating an important role of the endothelium in CVS pathophysiology [25]. The current study investigates other factors involved in vascular homeostasis.

The primary hypothesis was that the angiopoietin system is altered in patients developing severe vasospasm and radiographic infarcts after SAH. Therefore, Ang-1 and Ang-2 serum concentrations were longitudinally measured in SAH patients monitored for the occurrence of CVS and DCI.

## Methods

### Study Population

Between November 2007 and January 2009 twenty consecutive patients with aneurysmal SAH admitted to the neurocritical care unit of the Department of Neurology of Innsbruck Medical University were enrolled in this prospective study. All patients were treated by endovascular coiling with electrolytically detachable platinum coils, six patients (30%) received additional vascular stents. The study protocol was approved by the Ethics Committee of Innsbruck Medical University (Reference Number UN3021, 256/4.17). Inclusion criteria: SAH confirmed by cerebral computed tomography (CT), ruptured intracranial aneurysm demonstrated by digital subtraction angiography (DSA) for which interventional coiling was possible, first signs and symptoms having occurred within 48 hours before screening, written informed consent before recruitment or at time of regaining consciousness and WFNS grades I-V. Exclusion criteria: intracerebral or intraventricular blood without aneurysmal bleeding source, moderate to severe vasospasm at screening angiography, known coagulopathies, treatment with thrombocyte aggregation inhibitors or vitamin-K antagonists and severe pre-existing concomitant diseases.

Twenty age and gender matched healthy volunteers were recruited from hospital workers and relatives of the study investigators (mean age: 52.2, range: 33-68). All data was analyzed on an intention-to-treat basis.

### Sample collection and measurement

Blood samples of SAH patients were prospectively collected daily for the first 7 days, then every other day until 15 days post SAH. The first sample was taken before DSA was performed. Single blood samples from 20 age and gender matched volunteer donors served as healthy controls. Blood was collected using Sarstedt Monovette serum tubes. After at least 30 minutes of clotting time serum was obtained by centrifugation at 1500 rcf for 15 min within two hours after blood collection and stored at -80°C until use. Ang-1 and Ang-2 were measured in serum samples using enzyme-linked immunosorbent assay (R&D Systems, Minneapolis, MN) according to the manufacturer's instructions.

### Transcranial Doppler sonography (TCD) and patient management

TCD was performed daily from day 1 to 7 and every other day thereafter. Recordings of the mean blood flow velocities (mBFV) were performed through the trans-temporal ultrasound window using a 2-MHz handheld transducer probe (Compumedics DWL Multidop X4, Melbourne, Australia) when pCO_2 _levels where within normal ranges. Doppler sonographic cerebral vasospasm (dCVS) was defined as mBFV of 120 cm/s or more in the middle cerebral artery [26]. DCI was defined as new infarct on CT scan that had not been detected on the admission or the immediate post-interventional scan, and that was classified as vasospasm related by the research team. Other potential causes of CT pathologies, (e.g. rebleeding, cerebral edema or ventriculitis) were excluded. CT scans were also performed at discharge and were assessed by an independent radiologist.

At the end of hospitalization and after 6 months outcome was evaluated by modified Rankin Scale (mRS) and the Glasgow Outcome Scale (GOS). Demographic, clinical and laboratory values were recorded prospectively throughout the study. Patients experiencing dCVS received hemodynamic augmentation involving a target central venous pressure of > 8 mm Hg according to local protocols, which have been published previously [2]. Hypertension was induced using norepinephrine or phenylephrine infusion and fluid to maintain a mean arterial blood pressure of ≥100 mmHg. All patients received nimodipine either per os or intravenously at a daily dose of 300 mg, unless hemodynamic instability or hypotension occurred.

### Statistical methods

Angiopoietin levels were compared between the patient groups by Wilcoxon rank-sum test or Wilcoxon signed-rank test, as appropriate. The false discovery rate (FDR) criterion was used for controlling the errors in multiple comparisons [27]. To test the association between cerebral vasospasm and levels of Ang-1 and Ang-2 for important covariates (age, sex, white blood cell count (WBC), C-reactive protein (CRP) and body temperature), generalized estimation equations (GEE) were calculated with day post SAH and presence of dCVS as factors. To avoid co-linearity five different models were calculated, one for each of the respective covariates. Ang-1 and Ang-2 values were transformed logarithmically for this approach. Data are presented as mean ± SEM unless otherwise stated. Calculations were done using the PASW 18 (SPSS Inc., Chicago, IL, USA). Graphs were drawn with GraphPad Prism 5.00 software (GraphPad Prism Software Inc., San Diego, CA, USA).

## Results

### Patients' characteristics

Patients' age ranged from 31 to 66 years (mean 52.2 years), 4 patients were of male gender, 16 were female. One patient showed mild vasospasm during intervention on day one. Another ten patients developed dCVS between day 2 and 13 (1 patient on day 2, 4 patients on day 4, 3 patients on day 6, 1 patient on day 11 and 1 patient on day 13). Seven patients developed cerebral ischemia attributable to vasospasm (CIV). Demographic, clinical and laboratory characteristics of all patients are listed in table 1 and were compared based on the presence of dCVS. Baseline characteristics were comparable between both groups.

**Table 1:** Baseline characteristics including demographic and laboratory data of the study population

Parameter	dCVS absent	dCVS present	p-value
**number of patients**	9	11	
**age (mean, range)**	52.6 (31-66)	51.9 (39-64)	0.655*
**female gender, n (%)**	8 (88.9%)	8 (72.7%)	0.369†
**WFNS scale, n (%)**			0.119†
**I**	4 (44.4%)	3 (27.3%)	
**II**	0 (0%)	4 (36.4%)	
**III**	2 (22.2%)	0 (0%)	
**IV**	1 (11.1%)	3 (27.3%)	
**V**	2 (22.2%)	1 (9.1%)	
**Fisher grade, n (%)**			0.243†
**II**	2 (22.2%)	0 (0%)	
**III**	2 (22.2%)	4 (36.4%)	
**IV**	5 (55.6%)	7 (63.6%)	
**mRS on discharge**			0.545†
**0**	3 (33.3%)	4 (36.4%)	
**1**	3 (33.3%)	1 (9.1%)	
**2**	0 (0%)	1 (9.1%)	
**3**	1 (11.1%)	3 (27.3%)	
**4**	0 (0%)	1 (9.1%)	
**5**	1 (11.1%)	0 (0%)	
**6**	1 (11.1%)	1 (9.1%)	
**Length of stay in days, mean (range)**	20.3 (9-41)	25.5 (12-53)	0.394*
**Occlusive hydrocephalus requiring EVD**	3 (33.3%)	7 (63.6%)	0.178†
**Ventriculo-peritoneal shunt**	1 (11.1%)	1 (9.1%)	0.881†
**Intracerebral hemorrhage**	3 (33.3%)	3 (27.3%)	0.769†
**Cerebral edema**	5 (55.6%)	8 (72.7%)	0.423†

dCVS: Doppler sonographic cerebral vasospasm, WFNS: World federation of neurosurgical societies, mRS: modified Rankin scale, EVD: external ventricular drainage, Ang-1: angiopoietin-1, Ang-2: angiopoietin-2. * Analysis of variance, † chi-square test.

### Time course of Ang-1 and Ang-2 serum levels

**Figure 1:**
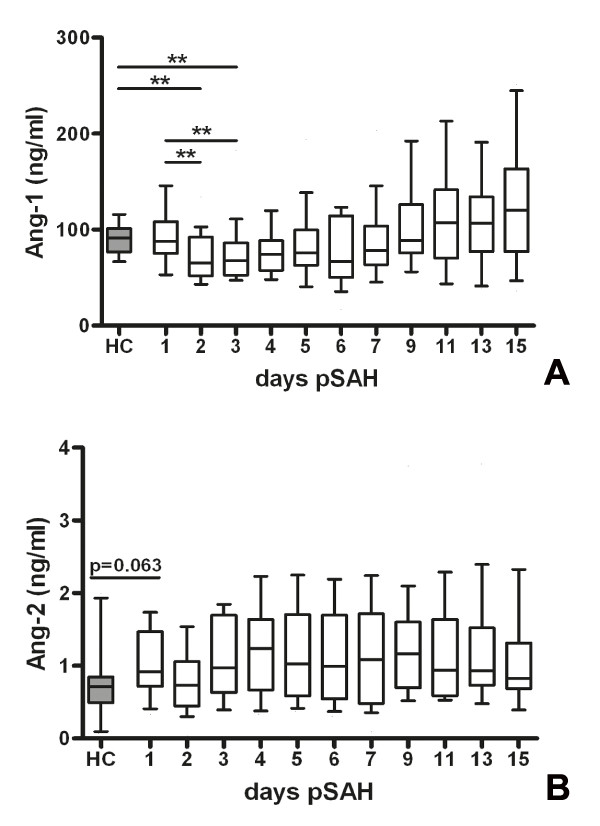
**(A) Angiopoietin-1 (Ang-1, ng/ml) and (B) angiopoietin-2 (Ang-2, ng/ml) serum concentrations in patients with aneurysmal subarachnoid hemorrhage (white boxes) and healthy control subjects (grey box)**. Wilcoxon rank-sum test for comparisons of HC with SAH patients, Wilcoxon signed-rank test for comparisons of the respective days among SAH patients. P-values adjusted for multiple comparisons by the FDR-method. **, p < 0.01.

Ang-1 levels decreased significantly on day 2 and 3 compared to baseline (p < 0.05, figure 1a). Ang-1 levels on days 2 and 3 also differed significantly between SAH patients and healthy controls (p < 0.05). Compared to day 2, when Ang-1 levels reached lowest values, there was a significant increase starting on day 5 reaching levels comparable to healthy controls (p < 0.05 for days 5 and 7, p < 0.01 for days 9, 11, 13 and 15).

Ang-2 levels did not differ significantly between days (figure 1b) or between patients and healthy controls. There was a trend towards higher Ang-2 serum concentrations in SAH patients on day 1 compared to healthy controls (p = 0.063).

**Figure 2:**
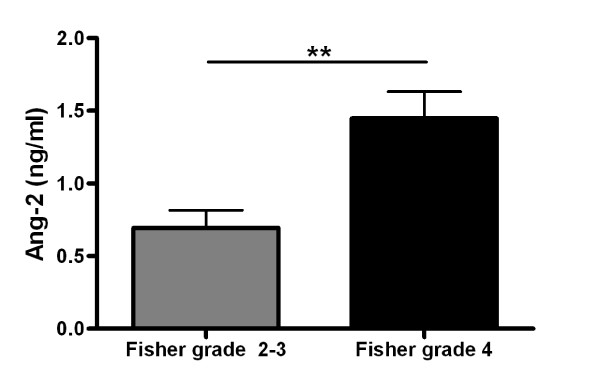
**Angiopoietin-2 (Ang-2, ng/ml) serum levels in patients with Fisher grade 2 and 3 compared to Fisher grade 4**. Mean and SEM. Wilcoxon rank-sum test. **, p < 0.01.

Ang-2 levels were significantly higher in patients with Fisher grade 4 compared to patients with Fisher grade 2 and 3 (p < 0.01, figure 2). Neither Ang-1 nor Ang-2 levels differed significantly between patients receiving additional vascular stents and patients without stents (data not shown).

### Doppler sonographic cerebral vasospasm

**Figure 3:**
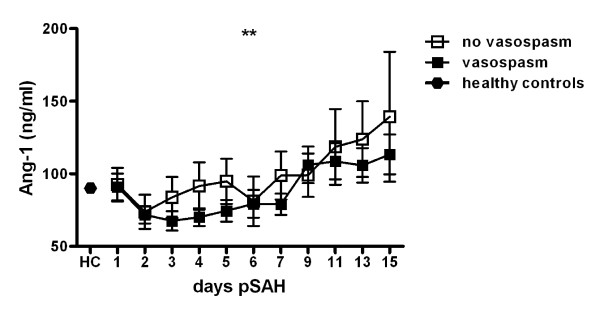
**Time course of angiopoeitin-1 (Ang-1, ng/ml) in patients with Doppler sonographic cerebral vasospasm (dCVS) compared to patients without dCVS**. Mean and SEM. Data of SAH patients modeled by generalized estimation equations with day post SAH and presence of dCVS as factors. **, p < 0.01.

To analyze the time course of angiopoietin levels and its association to the development of dCVS, multivariate generalized estimation equations were applied with day post SAH and presence of dCVS as factors including important covariates (age, sex, WBC, CRP and body temperature). These models showed a statistically highly significant effect of the interaction of both factors indicating different dynamics for Ang-1 in patients with or without dCVS, respectively (p < 0.001, figure 3). In contrast to patients without dCVS, in whom Ang-1 increased earlier starting from day 3, patients suffering from dCVS showed a delayed increase of Ang-1 serum concentrations.

For Ang-2 serum levels no significant association to dCVS was found (data not shown).

### Cerebral ischemia attributable to vasospasm

**Figure 4:**
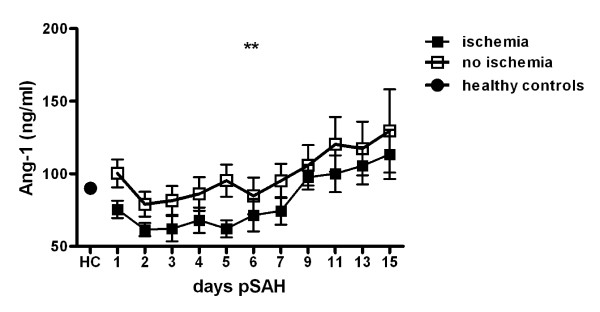
**Time course of angiopoietin-1 (Ang-1, ng/ml) in patients with cerebral ischemia attributable to vasospasm (CIV) compared to patients without CIV**. Mean and SEM. Data of SAH patients modeled by generalized estimation equations with day post SAH and presence of CIV as factors. **, p < 0.01.

Ang-1 serum levels on day 1 were significantly lower in patients who developed CIV (n = 7) compared to patients without CIV in the later course of SAH (n = 13) (Wilcoxon rank-sum test, p < 0.05). The GEE models could verify a time dependent difference of Ang-1 in patients with and without CIV showing a highly significant effect of the interaction of factors CIV and day post SAH, independent of the above mentioned covariates (p < 0.001; figure 4).

For Ang-2 serum levels no significant association to CIV could be found (data not shown).

## Discussion

This pilot study describes the time course of Ang-1 and Ang-2 serum levels in patients with aneurysmal SAH and their association with the development of dCVS and CIV. Our main findings were: 1) Ang-1 serum concentrations were significantly lower in SAH patients on days 2 and 3 compared to baseline levels, 2) serum levels of Ang-1 differed significantly between SAH patients and healthy controls, 3) patients with dCVS, and in particular patients with CIV secondary to dCVS, revealed a different time course of Ang-1 serum concentrations with delayed recovery of low Ang-1 levels observed in the early course of the disease.

The vascular endothelium modulates vascular tone through the release of various vasoactive substances regulating smooth muscle cell contraction [28]. A sensitive equilibrium between vasoconstricting and vasorelaxing substances is crucial for the maintenance of normal (blood) vessel diameter [28]. CVS is characterized by a prolonged and enhanced contraction of smooth muscle cells in the arterial vessel wall [28]. Amongst others it is caused by calcium-dependent vasoconstriction, upregulation of vasoconstrictors and decreased levels of vasorelaxing substances such as Endothelin-1 (ET-1) and nitric oxide (NO), respectively [28]. ET-1 is the most important endothelial factor mediating vasoconstriction and is up-regulated during CVS [29]. Interestingly, Ang-1 down-regulates the expression of ET-1 *in vitro *reducing ET-1 mRNA and protein levels [30]. Results from animal experiments show reduced ET-1 after injection of Ang-1 transfected fibroblast cells in the rat lung [30]. Moreover, it was shown that Ang-1 up-regulates the endothelial nitric oxide synthase, an important source of vasorelaxant NO. In our study, Patients with dCVS, and in particular with CIV, revealed a delayed increase of Ang-1 and showed lower values of Ang-1. It is tempting to speculate that a lack of Ang-1 contributes to outbalanced vasoconstrictive substances such as ET-1.

Another important feature of CVS is endothelial cell apoptosis [31,32]. Cerebral endothelial cell death has been reported after SAH in rats [33]. Apoptosis of endothelial cells has been suggested to expose smooth muscle cells within the vessel walls to damaging and vasoconstrictive substances within the blood flow [32]. The regulation of endothelial cell viability is a crucial function of angiopoietins with Ang-1 ensuring endothelial survival and Ang-2 inducing endothelial cell death [9,10]. We found decreased levels of Ang-1, an anti-apoptotic factor on endothelial cells, in patients with dCVS. This could further support the importance of endothelial apoptosis in the pathogenesis of CVS after SAH.

Other markers for vascular injury are endothelial microparcticles, which have been recently found to be associated with dCVS and CIV by our study group [25]. Ang-1 has been shown to suppress the generation of endothelial microparticles *in vitro *[34]. Lower Ang-1 levels might explain the increased levels of endothelial microparticles observed in patients with dCVS and CIV.

Data from various experimental and clinical studies suggest that Ang-1 is protective in cerebral ischemia. In the acute phase after ischemic stroke, Ang-1 is regarded as a protective factor on the vascular endothelium with important functions regarding blood-brain barrier stability [35]. This is supported by the fact that reduced levels of Ang-1 after cerebral ischemia are associated with blood-brain barrier breakdown [36]. The application of COMP-Ang-1, a soluble Ang-1 variant, in rats induced reduction of infarct volume and neurological deficits [37]. Zhao and colleagues report a protective effect of Ang-1 in a rat model of cerebral ischemia [36]. We found decreased levels of Ang-1 in patients with CIV. This further supports a possible protective role of Ang-1 in ischemic brain damage. Importantly, Ang-1 levels in patients with dCVS differed starting on day 3, whereas patients with CIV revealed different Ang-1 levels from the very first day. Pathologic alterations, such as acute CVS, cytotoxic edema and metabolic changes, have been described immediately after experimental and clinical SAH [38]. The current findings further corroborate the idea that mechanisms triggered by the initial bleeding are determining the predisposition for delayed cerebral infarction. Difference in baseline Ang-1 might reflect early impairment of vascular function in those patients, who develop symptomatic vasospasm later on. Surprisingly, we found significant alterations of Ang-1 associated with dCVS but not with Ang-2. Ang-1 is a product of pericytes, smooth muscle cells and fibroblasts, in contrast to Ang-2, which is mainly expressed by endothelial cells [9,10]. This might suggest a predominant role of perivascular cell types in the pathogenesis of CVS. However, further studies are required to evaluate the preponderance of endothelial or smooth muscle cell derived mechanisms respectively in the pathophysiology of cerebral vasospasm during SAH.

It should be noted that in the current study the diagnosis of CVS was based on TCD evaluations and not on digital subtraction angiography. Although the observed incidence of dCVS was within the known ranges [6] we might have missed some patients with CVS since the sensitivity of TCD in detecting angiographic CVS in not 100% [39,40]. However, analyzing patients with cerebral ischemia also revealed a significant change in the time course of Ang-1 serum concentration indicating that Ang-1 alterations occur in both dCVS and CIV. Though not necessarily associated with clinical symptoms/neurologic deficits, transient changes in cerebral vasculature, i.e. dCVS, seem to alter the release of Ang-1 from perivascular cells.

Our study was designed as a pilot study and therefore only included a small number of patients, which might be regarded as a limiting factor. Importantly, patients were well matched and showed a representative distribution of demographic and clinical characteristics. In addition the incidence of CVS, DCI and mortality was similar to previously local and international published data [6,41,42].

## Conclusions

In summary, this is the first report of the temporal dynamics of Ang-1 and Ang-2 during the course of spontaneous subarachnoid hemorrhage. Ang-1 levels showed an initial decrease after ictus and a delayed return to baseline values in patients who developed dCVS in the course of the disease. In patients suffering from CIV, lower values of Ang-1 have already been observed on the day of admission. Ang-1 is likely to play an important role in SAH pathophysiology and in the development of CVS. Its exact function in this regard as well as potential therapeutic implications warrant further investigation.

## Competing interests

The authors declare that they have no competing interests.

## Authors' contributions

PL, RB and AD coordinated the study. MF and AD carried out the immunoassays. PL and MF performed the statistical analysis. PL, RB, RH, BP, AC and ES collected clinical data and blood samples, evaluated outcome and performed neurologic examination. PL, RB and ES were responsible for designing the study. MF, GB and PL drafted the first version of the manuscript. All authors read, revised and approved the final version.

## Pre-publication history

The pre-publication history for this paper can be accessed here:

http://www.biomedcentral.com/1471-2377/11/59/prepub
